# USING OBSERVATIONAL DATA TO INFORM CANDIDATE PHENOTYPES FOR GEROSCIENCE TRIALS

**DOI:** 10.1093/geroni/igz038.2729

**Published:** 2019-11-08

**Authors:** Jason L Sanders, Alice Arnold, Robert Boudreau, Stephen Kritchevsky, Anne Newman

**Affiliations:** 1 Brigham and Women’s Hospital, Boston, Massachusetts, United States; 2 University of washington, Seattle, Washington, United States; 3 University of pittsburgh, Pittsburgh, Pennsylvania, United States; 4 wake forest school of medicine, Winston-Salem, North Carolina, United States

## Abstract

Geroscience trials will manipulate aging mechanisms which may have pleiotropic effects and alter multiple biologic processes and clinical outcomes. Determining an intervention’s efficacy and safety will require measuring several aspects of aging and intermediate endpoints with less regard to specific diseases. Picking the right measurements will significantly impact a trial’s cost-effectiveness and chance of success. Observational studies are ideal resources to test candidate phenotypes before investing in trials. We present a decade’s worth of results from the Cardiovascular Health Study as examples of using observational data to inform measurement in geroscience trials. Specifically, we illustrate the underlying theory, construction, operational characteristics, and inter-relationships of candidate phenotypes spanning circulating biomarkers, tissue and organ structure, and functional status, all of which can be used in geroscience trials depending on the intervention’s target and predicted outcome.

